# Assessing effectiveness of heart rate variability biofeedback to mitigate mental health symptoms: a pilot study

**DOI:** 10.3389/fphys.2023.1147260

**Published:** 2023-05-10

**Authors:** Thais Castro Ribeiro, Pau Sobregrau Sangrà, Esther García Pagès, Llorenç Badiella, Beatriz López-Barbeito, Sira Aguiló, Jordi Aguiló

**Affiliations:** ^1^ Biomedical Research Network Center in Biogineering, Biomaterial and Nanomedicine (CIBER-BBN), Madrid, Spain; ^2^ Department of Microelectronics and Electronic Systems, Autonomous University of Barcelona, Barcelona, Spain; ^3^ Clínic Foundation for Biomedical Research, Hospital Clínic of Barcelona, Barcelona, Spain; ^4^ Applied Statistics Service, Autonomous University of Barcelona, Barcelona, Spain; ^5^ Emergency Department, Hospital Clínic of Barcelona, Barcelona, Spain

**Keywords:** stress objective assessment, effectiveness of interventions, electrophysiological model, heart rate variability biofeedback, healthcare workers, mental health, stress

## Abstract

**Introduction:** The increasing burden on mental health has become a worldwide concern especially due to its substantial negative social and economic impact. The implementation of prevention actions and psychological interventions is crucial to mitigate these consequences, and evidence supporting its effectiveness would facilitate a more assertive response. Heart rate variability biofeedback (HRV-BF) has been proposed as a potential intervention to improve mental wellbeing through mechanisms in autonomic functioning. The aim of this study is to propose and evaluate the validity of an objective procedure to assess the effectiveness of a HRV-BF protocol in mitigating mental health symptoms in a sample of frontline HCWs (healthcare workers) who worked in the COVID-19 pandemic.

**Methods:** A prospective experimental study applying a HRV-BF protocol was conducted with 21 frontline healthcare workers in 5 weekly sessions. For PRE–POST intervention comparisons, two different approaches were used to evaluate mental health status: applying (a) gold-standard psychometric questionnaires and (b) electrophysiological multiparametric models for chronic and acute stress assessment.

**Results:** After HRV-BF intervention, psychometric questionnaires showed a reduction in mental health symptoms and stress perception. The electrophysiological multiparametric also showed a reduction in chronic stress levels, while the acute stress levels were similar in PRE and POST conditions. A significant reduction in respiratory rate and an increase in some heart rate variability parameters, such as SDNN, LFn, and LF/HF ratio, were also observed after intervention.

**Conclusion:** Our findings suggest that a 5-session HRV-BF protocol is an effective intervention for reducing stress and other mental health symptoms among frontline HCWs who worked during the COVID-19 pandemic. The electrophysiological multiparametric models provide relevant information about the current mental health state, being useful for objectively evaluating the effectiveness of stress-reducing interventions. Further research could replicate the proposed procedure to confirm its feasibility for different samples and specific interventions.

## 1 Introduction

In recent years, mental health has become a global concern. The increasing prevalence of mental health disorders also impairs physical health, causing disability and negative economic and social impacts (Flores et al., 2018). The World Health Organization (WHO) recognizes that mental healthcare is an essential part of comprehensive care, thus supporting the need for actions to promote mental wellbeing and implement prevention interventions (World Health Organization, 2022). There is substantial literature regarding psychological interventions (Purtle et al., 2020), although there is scarce evidence supporting their validity, and limited quantitative outcomes have been considered (Ahmed et al., 2021; Cairns et al., 2021).

To advance in the field of mental health, it is crucial to establish reliable tools that allow to objectively assess the patient’s affectability and the effectiveness of applied interventions. The measurement of electrophysiological variables provides information about the functioning of the autonomic nervous system (ANS) and permits estimating the stress level non-invasively, being an extended quantitative approach for this purpose (Ahmed et al., 2022).

A sustained stress response generates an ANS imbalance as the body tends to hyperactivate the sympathetic nervous system (SNS) and the hypothalamus–pituitary–adrenal axis (HPA), leading to hormonal and physiological adaptations for a “fight-or-flight response.” The parasympathetic nervous system (PNS) typically facilitates the recovery of stress responses, giving flexibility to adapt to different daily situations, although under a chronic stress condition, this function is compromised (McCraty and Shaffer, 2015).

Cardiac function is strongly regulated by the brain through dynamic interactions between the SNS and PNS (Tonhajzerova et al., 2012). Heart rate variability (HRV), which reflects the fluctuation in time intervals between adjacent heartbeats, and its metrics have become widely used indices of these interactions, and hence, of psychological wellbeing. Low HRV has been associated with a diminished regulation capability of the ANS, cardiovascular outcomes, impairment of the immune system, aging, and other conditions. Moreover, high HRV has been linked with better performance, health, and adaptability (Kim et al., 2018). Nevertheless, evidence showed limitations when interpreting HRV parameters in a simplistic binary way (sympathetic and parasympathetic) and highlighted the complexity of this system and influencing factors, such as age and respiratory rate (Billman, 2013; Hernando et al., 2016; Hayano and Yuda, 2019).

In the literature, the outcomes more frequently selected to evaluate the effect of mental health interventions are as follows: a) psychometric questionnaires to subjectively estimate the anxiety state, stress level, and severity of depression, among others (Lehrer et al., 2020); b) HRV parameters to assess ANS functioning (Heiss et al., 2021); and c) electrodermal activity (EDA) indexes to evaluate sympathetic activation (Scavone et al., 2020). We hypothesize that use of subjective measures and single-objective indices limits the understanding of the stress response; hence, a multimodal approach is being proposed in the current study. Indices derived from electrocardiogram (ECG), EDA, peripheral skin temperature and respiration collectively may provide a more comprehensive information on physiological behavior and stress response (Garzón-Rey, 2017; Arza et al., 2018; García Pagès et al., 2023). Our research group has suggested combined models that include a set of electrophysiological variables, which were constructed from statistical models based on the usual clinical psychological assessment (self-reported questionnaires), thus providing a more robust estimation (Garzón-Rey et al., 2017; Aguiló Mir et al., 2021).

The heart rate variability biofeedback (HRV-BF) is a proactive intervention that allows easy multimodal signal recording and quantitative measurements, besides being broadly applied for mental health issues (Moss and Shaffer, 2017). One of the HRV-BF mechanisms is based on the physiological relation between cardiovascular and respiratory systems, which is mediated by the vagus nerve, also known as respiratory sinus arrhythmia (RSA), where the heart rate accelerates during inhalation and decelerates during exhalation. Breathing at a certain respiratory rate—in general between 4.5 and 6.5 cycles per minute (resonance frequency)—stimulates the cardiovascular system’s resonant properties, producing larger oscillations of heart rate, leading to higher HRV in individuals and other beneficial effects, such as better gas exchange (Yasuma and Ichiro Hayano, 2004).

The other mechanism of HRV-BF is the stimulation of the baroreflex, which is also vagally regulated and responsible for blood pressure (BP) homeostasis. Upon an increase in BP, the baroreflex induces a rapid adjustment in heart rate, reducing the blood flow and, subsequently, lowering BP (Lehrer, 2013). Again, when breathing at the resonance frequency, the RSA stimulates the baroreflex, thus strengthening its sensitivity. Therefore, HRV-BF training stimulates parasympathetic activity through both RSA and baroreflex, thus enhancing autonomic functioning (Lehrer, 2013).

The HRV-BF applications are diverse; importantly, it has been considered an effective intervention to reduce stress and anxiety (Yu et al., 2018; Kennedy and Parker, 2019), to mitigate posttraumatic stress symptoms (Schuman and Killian, 2019), and to reduce symptoms of depression (LinMei et al., 2019; Pizzoli et al., 2021). It was also proposed as a promising intervention to reduce physiological distress in healthcare workers (HCWs) in the COVID-19 pandemic (Aristizabal et al., 2020); however, there is no evidence in this context yet. Seeking to fill this gap and considering that frontline HCWs constitute a vulnerable group, especially with the emergence of the COVID-19 pandemic (Giorgi et al., 2020; Pappa et al., 2020; Sobregrau Sangrà et al., 2022), the current study applied an HRV-BF protocol in this population.

The main aim of this study is to propose and validate an objective procedure to easily assess the effectiveness of mental health interventions, specifically in this study of a personalized HRV-BF protocol in mitigating mental health symptoms in frontline HCWs who worked in the COVID-19 pandemic. For this purpose, we designed a PRE and POST experimental study based on two different approaches of psychological assessments. First, considering the gold-standard psychometric questionnaires and second, applying a recently developed electrophysiological multiparametric model that has been validated to assess chronic stress levels. As a secondary objective, we propose to investigate whether an acute stress model can distinguish the different stages of physiological response assessment (baseline, stress exposure, and recovery), and also the most relevant variables for this purpose in this population.

## 2 Methods

### 2.1 Participants

Twenty-one frontline HCWs were included in this study between March and July 2021, recruited from two Spanish tertiary hospitals: Hospital Clínic of Barcelona and Hospital del Mar of Barcelona. The study was conducted at the Hospital Clínic of Barcelona, and the protocol was approved by its Ethics Committee. All participants gave a written informed consent.

The inclusion criteria were to be a HCW directly involved with care of COVID-19 patients during the pandemic. The selection was made according to a high risk of developing post-traumatic stress disorder or a moderate perception of stress and/or anxiety according to cutoff points of current gold-standard questionnaires (i.e., a score of 30 or higher on PCL5; 14 or higher on PSS; and 10 or higher on STAI-S) and voluntarily agreeing to participate.

### 2.2 Experimental procedure

**FIGURE 1:**
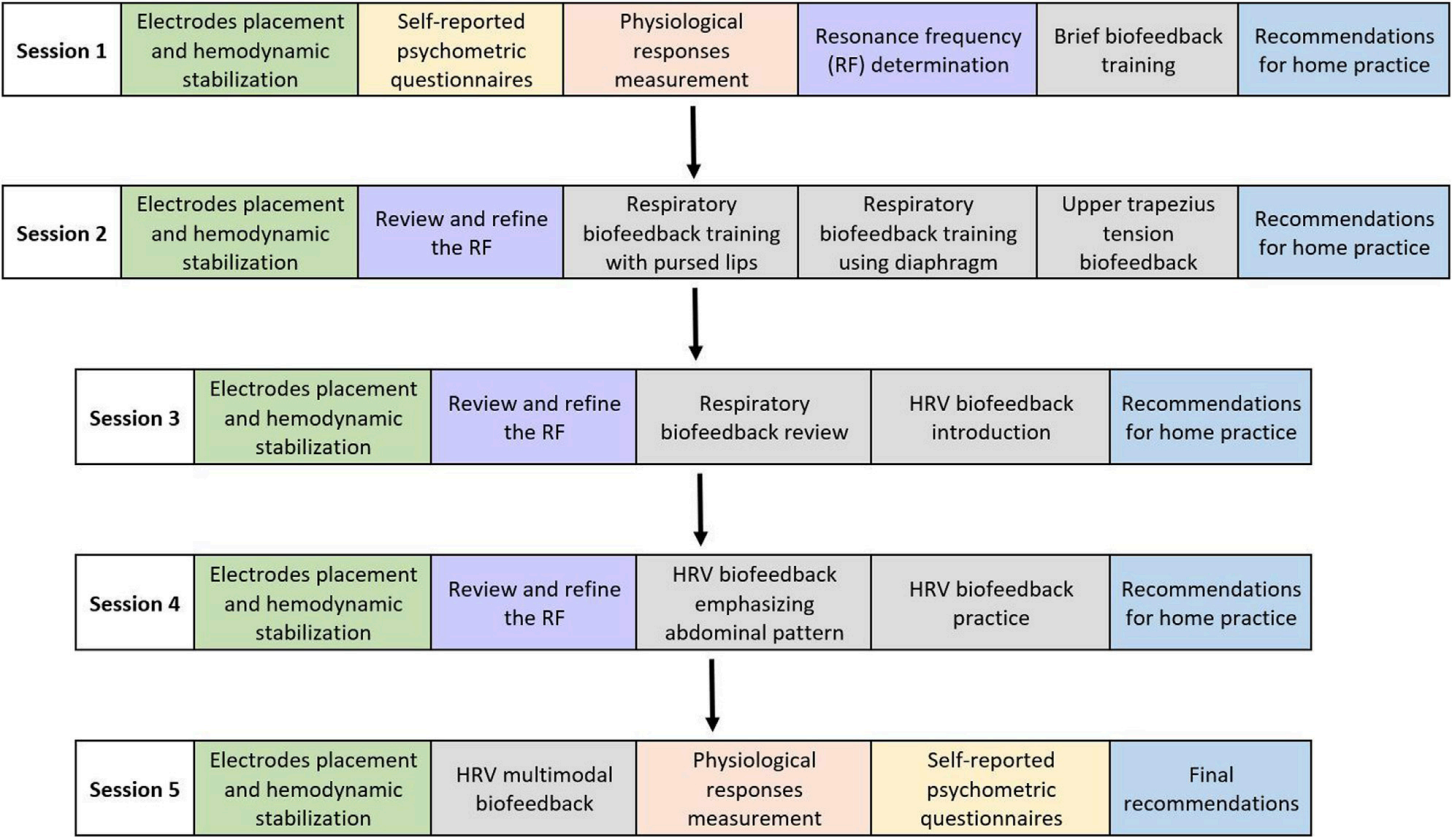
Scheme of the 5-session experimental protocol. The colors identify the different steps in each of the sessions. Those marked in gray represent the biofeedback training.

The HRV-BF protocol was designed based on (Lehrer et al., 2013) and comprises 5 weekly in-person sessions of 45–60 min, including specific instructions for daily practices. Each session was held individually in a quiet room conducted by a trained professional and scheduled according to participant availability. If a participant could not attend one of the sessions, it was rescheduled for as soon as possible in order to complete the protocol and to prevent a longer interval between sessions from compromising adherence to practice. Figure 1 shows the organization of the overall protocol by sessions.

Participants provided information on demographics, working conditions, psychological background, use of medication, and social habits. Self-reported psychometric questionnaires (yellow blocks shown in Figure 1) were collected, and a physiological response measurement (salmon-colored blocks) was carried out prior to (PRE) and immediately after (POST) the HRV-BF training to perform comparisons.

In the first session, preceding the training, the individual’s resonance frequency (RF), i.e., the optimal breathing rate, was determined based on the following criteria: phase synchrony between respiration and HR and signal smoothness, maximal HRV (higher peak–trough amplitude), greater power in low-frequency (LF) band, and a peak around 0.1 Hz on the LF band (Lehrer et al., 2013; Shaffer and Meehan, 2020). RF was revised and fine-tuned in the following sessions (lilac-colored blocks).

In sessions 2, 3, 4, and 5, the biofeedback training (gray blocks) was focused on specific breathing techniques, such as pursed lips and diaphragmatic breathing, guided by respiration and heart rate variability real-time waves displayed by using BioTrace + software (Mind Media, 2018 version). Participants were instructed to practice specific breathing exercises daily for 10–20 min (blue blocks). For home practice, the given recommendations increased the difficulty level at each session: week 1: starting with brief periods of slow breathing practice with a pacer to correctly follow the rhythm of the individual’s resonance frequency. The pace of breathing would become more fluid and comfortable for the participant, and the practice time would be gradually increased; week 2: same as the previous one and adding diaphragmatic breathing awareness exercises, such as placing one hand over the abdomen and the other over the chest and breathing in such a way that the hand over the abdomen moves and the other is as still as possible. It could be performed lying down to facilitate the perception of the movement; week 3: performing the same exercise as before without using the hands and more frequently in a sitting position. One must start practicing using the pacer and then performing brief periods of free practice; week 4: performing breathing without using the pacer whenever they feel safe following the already incorporated rhythm; then one must start applying it on day-to-day activities. In case of insecurity to do it freely, they should start the practice with the pacer and try to perform in short free periods and gradually increase the duration; week 5: it is recommended to continue performing slow breathing on a daily basis and applying it in everyday situations, especially after or before a stressful event. The importance of daily practice to achieve physiological and long-term benefits is emphasized to engage the participants. The use of free applications on their mobiles was encouraged to practice RF at home with a pacer (*Paced Breathing* for Android and *Awesome Breathing: Pacer Time* for iOS).

### 2.3 Study outcomes

#### 2.3.1 Self-reported psychometric questionnaires

Self-reported psychometric questionnaires consisted of gold-standard psychological questionnaires in validated Spanish versions to examine stress, anxiety, and depression under PRE–POST conditions. Stress was examined using the Perceived Stress Scale (PSS) (Remor, 2006) and the Visual Analog Stress Scale (VASS) (Lesage et al., 2012). The Posttraumatic Stress Disorder Checklist for DSM-5 (PCL5) was applied as a screening for post-traumatic stress disorder (PTSD) symptoms (Blevins et al., 2015). Anxiety symptoms were evaluated using the State-Trait Anxiety Inventory (STAI) (Spielberger et al., 1983), while to assess depression, the Patient Health Questionnaire-9 (PHQ9) was used (Diez-Quevedo et al., 2001). All instrument descriptions are given in Table 1.

**TABLE 1:** Self-reported instruments used to assess the outcomes.

Outcome	Instrument	Description
Stress perception	Visual Analog Stress Scale (VASS)	Visual 100-point scale (0, not at all; 100, absolutely stressed)
	Perceived Stress Scale (PSS)	10-item Likert scale (0, never; 4, very often)
Risk of PTSD	Post-traumatic Stress Disorder Checklist for DSM-5 (PCL-5)	20-item Likert scale (0, not at all; 4, extremely)
Current level of anxiety	State Anxiety Inventory (STAI-S)	40-item Likert scale (1, not at all; 4, very much so). Each subscale includes 20 items
Trait anxiety	Trait Anxiety Inventory (STAI-T)	
Depression	Patient Health Questionnaire-9 (PHQ9)	9-item Likert scale (0, not at all; 3, nearly every day)

The reliability of these questionnaires was assessed for the study sample by Cronbach’s alpha, and estimates were all above 0.85, thus showing a high degree of internal consistency (O’Rourke and Hatcher, 2013).

#### 2.3.2 Physiological variables

Physiological monitoring provided an insight into differences in autonomic functioning in a stress-inducing situation before and after the intervention. The participants were asked to be seated in a comfortable chair with armrests and eyes open. The physiological response measurement was analyzed in three different stages: 1) baseline measurement (BM), taken as a reference when the participant is in a resting state; 2) stress exposure (SE), to evaluate the physiological reactivity facing stressors; and 3) recovery measurement (RM) to assess resilience after stressors.

**FIGURE 2:**
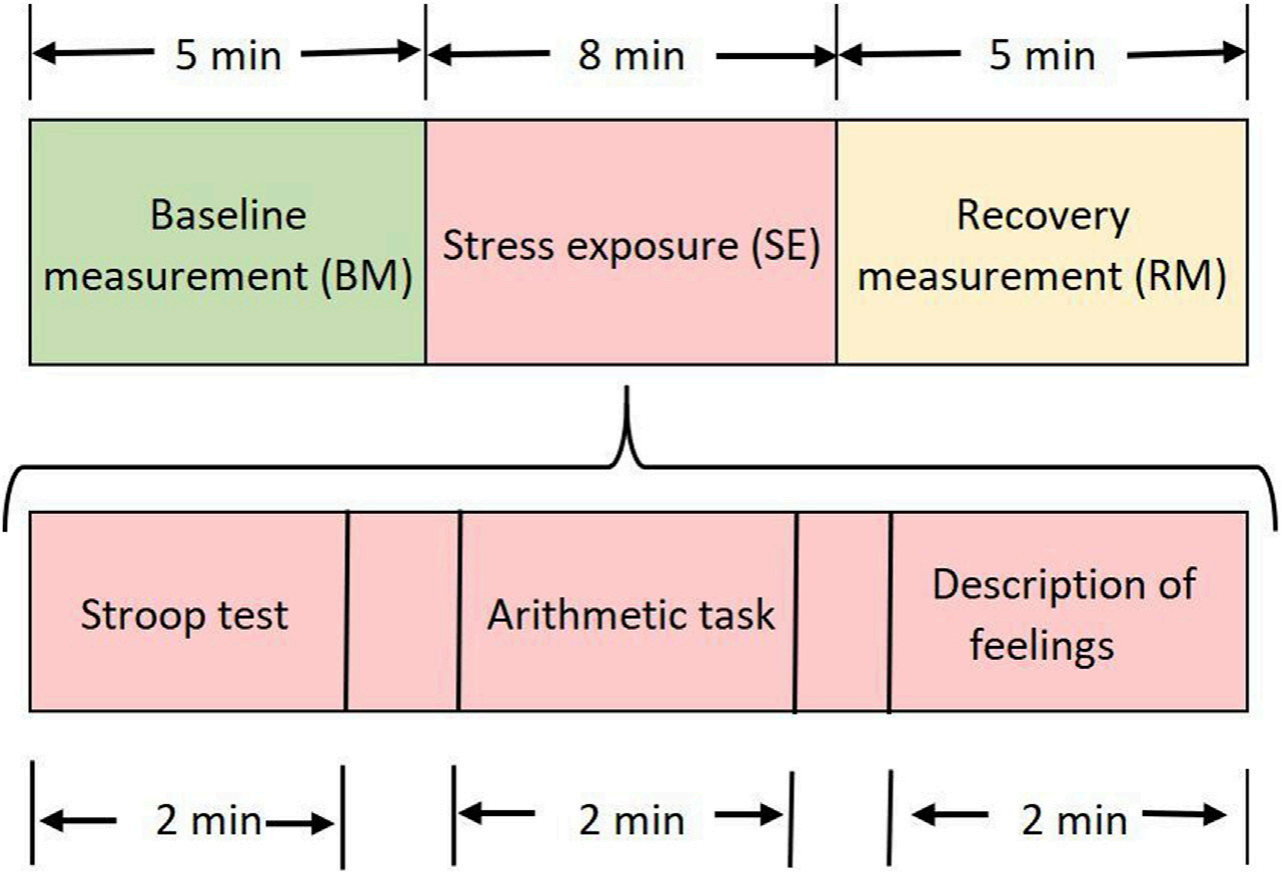
Schematic procedure for physiological response measurement PRE- and POST-intervention.

The SE stage included three different stressors: a) 2-min Stroop task (originally Stroop, 1935), with an incongruent stimulus, in which the colors were written in an ink that does not match the color-word, and the participant should inform the color of the ink; b) 2-min mental arithmetic task (Kirschbaum et al., 1993), which consisted of performing mental calculations as quickly and accurately as possible; in case of an error, the participant should start over; c) 2-min telling about the experience of working on the frontlines of the COVID-19 pandemic and the feelings they could report (detailed in Figure 2). Differences between stressors were not analyzed in this study.

##### 2.3.2.1 Data acquisition

The electrophysiological data were recorded using the NeXus-10 MKII device (Mind Media BV, Herten, Netherlands) and the real-time waves displayed by Biotrace+ software (Mind Media, 2018 version) during each of the sessions. Electrocardiogram (ECG), photoplethysmogram (PPG), electrodermal activity (EDA), skin temperature (ST), and respiration (Resp) were signals simultaneously gathered. The electromyogram (EMG) of the upper trapezius muscle was monitored only for bringing awareness of muscular activation during breathing, although this signal was not included in the study analyses.

For the EDA, disposable electrodes were affixed to the palmar surface of the middle phalanges of the second and fourth digits; for PPG, the clip sensor was placed on the middle finger, and on the fingertip of the fifth finger was attached the ST sensor. The electrode was placed in the non-dominant hand to avoid excessive movements and possible large artifacts.

For lead 1 of the ECG signal, electrodes were placed below the right collarbone and below the left rib cage, whereas for lead 2, electrodes were placed on the fifth intercostal space along the mid-axillary line on the left and the other symmetrically on the right side. The reference electrode was placed on the left collarbone.

To monitor the Resp signal, an adjustable elastic band was placed over the abdomen and over clothing. This sensor detects abdominal expansion/contraction; for instance, when inhaling, the device stretches, and during exhalation, the sensor returns to its neutral position.

Simultaneously, the PPG, EDA, and ST signals were monitored through the Empatica E4 wearable wristband (Empatica Boston, United States). These data will be explored in further analysis looking for a more easy and unobtrusive way to assess the effectiveness of interventions, considering the number of HRV-BF sessions and the interval between them.

##### 2.3.2.2 Data processing

The different electrophysiological raw signals mentioned were analyzed in a 1-min window using BioSig browser in MATLAB software (Garzón-Rey et al., 2017), and several variables were extracted from each one of them as described in Table 2. For comparisons, the mean of all 1-min segments was computed as a single value per stage for each subject.

**TABLE 2:** Description of extracted parameters from electrophysiological variables.

Electrophysiological signal	Sample frequency (Hz)	Extracted parameters	Description
Electrocardiogram (ECG)	1024	HRV-HR, bpm	Mean heart rate
		HRV-SDNN, s	Standard deviation of normal beat intervals
		HRV-RMSSD, s	Root mean square of successive differences between beat intervals
		HRV-sdsd, s	Standard deviation of differences between adjacent R–R peaks intervals
		HRV-VLF, s^-2^	Absolute power of the very low-frequency band (0.003–0.04 Hz)
		HRV-LF, s^-2^	Absolute power of the low-frequency band (0.04–0.15 Hz)
		HRV-HF, s^-2^	Absolute power of the high-frequency band (0.15–0.4 Hz)
		HRV-LF/HF ratio	Ratio of low-frequency to high-frequency power
		HRV-LFn, nu	Relative power of the low-frequency band normalized
		HRV-HFn, nu	Relative power of the high-frequency band normalized
Electrocardiogram (ECG) and photoplethysmography (PPG)	1024	PAT, ms	Mean pulse arrival time, the time between the beat detected by ECG and the pulse by PPG
		stdPAT, ms	Standard deviation of pulse arrival time
Respiration (Resp)	512	RR, Hz	Respiratory rate
		Pk, Hz	Respiratory rate peak in the power spectrum
Electrodermal activity (EDA)	512	Tonic, µS	Average value of the tonic component, i.e., slowly changing level of conductance of the skin, also known as skin conductance level (SCL)
		Phasic, µS	Average value of the phasic component, i.e., fast-changing responses typically associated with short-term events, also known as skin conductance responses (SCR)
		aucPhasic, µS·s	Area under the curve of the phasic component, related to SCR
		EDASymp, µS	Electrodermal response in the power spectrum (0.045–0.25 Hz)
Skin temperature (ST)	512	TFinger, °C	Mean finger temperature
		TGradient, °C	Mean gradient of finger temperature
		TPower, °C^2^	Mean power of finger temperature

In this study, the PPG signal will be used in conjunction with that of ECG to extract pulse arrival time (PAT) parameters. To extract HRV parameters, the ECG signal was chosen since it provides more accurate measurements.

From the ECG signal, the beat was detected through a discrete wavelet transform (Martínez et al., 2004). Given the robustness of the algorithm and the visual inspection of the quality of the ECG signals, no type of filtering was applied. Afterward, the existence of ectopic beats or false QRS detections was verified and fixed using the algorithm reported in Mateo and Laguna (2003) prior to the computation of the RR series. The algorithm searches for sudden changes in interbeat intervals, performs an interpolation where it has found non-normal beats, and based on this, proposes a correction to them. Segments of up to three interpolated/corrected beats have been accepted and are assumed to be normal. Following this, a time-domain analysis was performed to extract the mean heart rate (HRV-HR) in beats per minute; the standard deviation of differences between adjacent normal peak intervals (HRV-SDNN); and the root mean square of successive differences between beat intervals (HRV-RMSSD). For the frequency-domain analysis, the low- and high-frequency absolute powers (HRV-LF and HRV-HF, respectively) were computed from the spectral density of the HRV signal, calculated using Fourier transform. The HRV signal is obtained by subtracting from the instantaneous HR signal, a low-pass-filtered HR signal (cutoff frequency of 0.03 Hz), which mainly explains the changes in mean HR. The HRV–LF/HF ratio was computed by the ratio between absolute power in low- and high-frequency bands, i.e., HRV-LF/HRV-HF. The HRV-LFn and HRV-HFn represent the low- and high-frequency power normalized, respectively.

The PPG signal was filtered using a low-pass FIR filter with a cut-off frequency of 35 Hz (order 50) and, then, a high-pass FIR filter with a cut-off frequency of 0.3 Hz (order 5000). PPG artifacts were suppressed through a Hjorth-parameter-based PPG artifact detector described in Gil et al. (2009). Pulses were detected from the PPG signal on those time slots without artifacts by an algorithm based on Lazaro et al. (2014). Subsequently, the mean time difference between the R peak in the ECG signal and the point of 50% increase, corresponding to the pulse detected on the finger by the PPG signal, was considered the pulse arrival time (PAT), and its standard deviation (stdPAT) was also calculated.

The respiratory rate (RR) was estimated as the frequency to which the maximum peak (Pk) of the power density spectrum corresponds, estimated by means of fast Fourier transform (Lázaro et al., 2014). When the Pk was greater than 65%, then the RR was accepted as the respiratory rate. The respiration wave was filtered with an FIR passband filter with cutoff frequencies of 0.03 and 0.9 Hz. In 10 participants, the Resp parameters could not be calculated during the SE because they did not meet the condition aforementioned, probably due to the speech during the task, which affects the signal quality. The missing values were replaced by median imputation to perform the comparisons between stages.

The EDA signal was visually inspected to delete motion artifacts and linearly interpolated. The windows with interpolated segments larger than 25% were discarded, and the signal was then resampled at 4 Hz. Then, EDA was processed in two different ways. First, a time-domain analysis was conducted using a convex optimization model, called cvxEDA (Greco et al., 2016), which decomposes the signal as a linear combination of the tonic and phasic components (i.e., the skin conductance level and response, respectively) and some noise (incorporating the error of the model, artifacts, and other measurement errors). The average value of the tonic (Tonic) and phasic (Phasic) components and the area under the curve of the phasic component (aucPhasic) were calculated. The second way was a frequency-domain analysis of EDA, proposed to assess sympathetic tone through a parameter named EDASymp (Posada-Quintero et al., 2016). In this analysis, the EDA signal was filtered with an FIR bandpass filter with 0.01 and 0.9 Hz cutoff frequencies, and the power of the electrodermal response in the band 0.045–0.25 Hz (EDASymp) was computed. This bandwidth has been reported to correspond to sympathetic dynamics in non-exercise conditions.

Finally, for the skin temperature signal, visual inspection showed that the recorded signals did not contain significant artifacts; therefore, no pre-processing was needed. Apart from the skin temperature average value (TFinger), the gradient of the successive differences of TFinger every 10 s was calculated (TGradient), indicating the speed of temperature change. In relation to the amplitude, the power of the signal calculated as the mean of the square of the signal (TPower) is used.

#### 2.3.3 Multiparametric chronic and acute stress models

For a more robust estimation of the stress level, we used two different models a) to assess the chronic stress level (Aguiló Mir et al., 2021), called electrophysiological signal-based stress model (ESBSm) and b) to assess the acute stress level, called ES3 (Garzón-Rey, 2017). These models were calculated from the variables extracted from the electrophysiological signals explained in the previous section.

The chronic stress level model (ESBSm) was designed to match up with a reference scale including psychometric questionnaires and biochemical variables, taken as a gold-standard indicator of stress response. ESBSm includes 11 standardized parameters extracted from ECG, Resp, and EDA and a few quadratic terms. Some parameters are previously logarithmically transformed to avoid data skewness effects. In the present study, this model is applied as an instrument to assess the effectiveness of the HRV-BF protocol since it allows detecting changes in the chronic stress level over time.

The acute stress level model (ES3) also fits the results of psychometric questionnaires and biochemical biomarkers in a sample subjected to an induced acute stress task. The ES3 includes four parameters extracted from ECG, EDA, and ST. In the present study, ES3 is used to assess whether changes occur between the different stages (BM, SE, and RM). As with the previous model, the analysis was also performed comparing values in PRE and POST conditions.

### 2.4 Statistical analysis

A descriptive statistical analysis was performed to summarize the sample characteristics. Continuous variables are presented as mean and standard deviation (SD), and categorical variables are presented as frequency and percentages.

For the psychometric questionnaires and chronic stress model (ESBSm) data, differences in PRE–POST intervention were evaluated with paired *t*-tests or Wilcoxon signed-rank tests, according to data distribution.

For the electrophysiological variables, in order to compare conditions (PRE and POST) and stages (BM, SE, and RM), a mixed model (repeated measures analysis of variance, ANOVA) was used. The compliance of the application criteria was assessed with the analysis of model residuals. Skewed variables were analyzed using log-transformation.

Post-hoc tests with Tukey’s correction were performed to assess the time and stage effects on physiological variables and on the acute stress model (ES3). A *p* < 0.05 was considered statistically significant. Statistical analysis was performed using SAS^®^ v9.4 (SAS Institute, Cary, NC, United States).

## 3 Results

### 3.1 Sample characteristics

The study sample consisted of 21 female participants who completed the protocol in an average of 36 ± 11.4 days. The daily practice accomplishment was 62.28% (±19.1) regarding overall target home practice of 28 days. The mean age was 37.7 ± 11.7 years (range: 19–59 years), with an average body mass index of 24.2 ± 3.8 kg/m^2^. Most of the participants were nurses (61.9%), followed by physicians (28.6%) and auxiliary nurses (9.5%). All sociodemographic and clinical information is detailed in Table 3.

**TABLE 3:** Clinical and sociodemographic characteristics of participants.

Characteristics	Mean (SD)	N (%)
Age (years)	37.7 (±11.7)	
Protocol completion time (days)	36 (±11.4)	
Daily practice accomplishment (%)	62.3 (±19.1)	
BMI (kg/m^2^)	24.2 (±3.8)	
Normal (18.5–24.9)		12 (57.2%)
Overweight (≥25)		6 (28.6%)
Obesity (≥30)		3 (14.3%)
Physical activity		
Rarely		5 (23.8%)
Occasional		11 (52.4%)
Regular		5 (23.81%)
Increased substance use		
Yes		10 (52.4%)
No		11 (47.6%)
Working hours per week (hours)	38.3 (±3.6)	
Professional category		
Clinicians		6 (28.6%)
Nurses		13 (61.9%)
Auxiliary nurses		2 (9.5%)
Working unit		
COVID-19 wards		6 (28.6%)
Emergency service		13 (61.9%)
ICU		2 (9.5%)
Employment category		
Fixed-term		14 (66.7%)
Open-ended		7 (33.3%)
Living with people at high risk of COVID-19 infection		
Yes		7 (33.3%)
No		14 (66.7%)
Psychological support requested		
Yes		3 (14.3%)
No		18 (85.7%)
Working leave due to COVID-19		
Yes		11 (52.4%)
No		10 (47.6%)
Increase in weekly working hours		
Yes		9 (42.9%)
No		12 (57.1%)
Current stress-related medication		
Yes		4 (19%)
No		17 (81%)
Previous affective disorders		
Yes		7 (33.3%)
No		14 (66.7%)
Pharmacological treatment for stress		
Yes		16 (76.2%)
No		5 (23.8%)
Previous treatment with psychotropics		
Yes		4 (19%)
No		17 (81%)

Key: BMI, body mass index; ICU, intensive care unit; SD, standard deviation.

### 3.2 Primary outcomes

#### 3.2.1 Self-reported psychometric questionnaires

**FIGURE 3:**
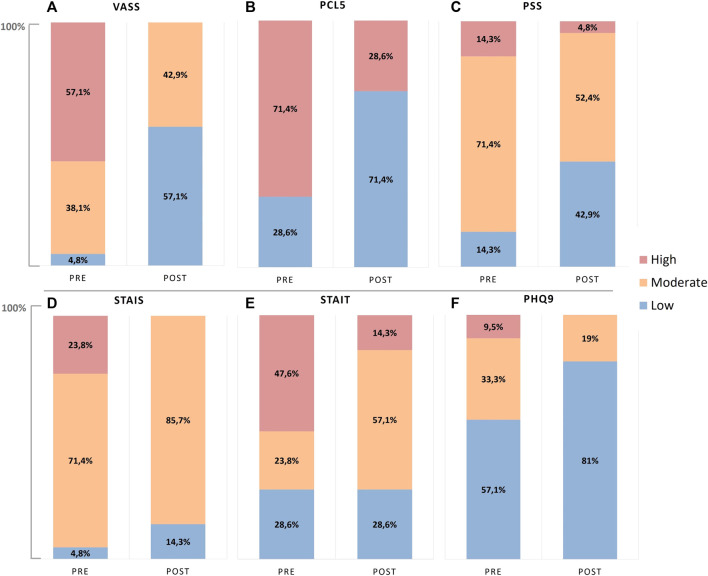
Severity classification of subjects based on the psychometrics cut-off points at PRE- and POST-intervention. **(A)** Stress perception with the Visual Analog Stress Scale (VASS), **(B)** risk to develop PTSD with Posttraumatic Stress Disorder Checklist for DSM-5 (PCL5), **(C)** perceived stress with the Perceived Stress Scale (PSS), **(D)** anxiety state with State Anxiety Inventory (STAI-S), **(E)** anxiety trait with Trait Anxiety Inventory (STAI-T), and **(F)** depression with Patient Health Questionnaire-9 (PHQ9).

Figure 3 shows the classification PRE- and POST-intervention of each psychometric questionnaire according to the cut-off points of previous studies (Aguiló Mir et al., 2021; Pedrozo-Pupo et al., 2020; Blevins et al., 2015; Kroenke et al., 2001). The classification is generally in three categories considering the degree of severity as low, moderate, or high, and in two categories for the PCL5 that classifies those with high or low risk of developing PTSD.

In the PRE-intervention condition, the study sample presented predominantly high anxiety trait scores (47.6%), moderate anxiety state (71.4%), and moderate perceived stress (71.4%) and showed increased risk of developing PTSD (71.4%). Regarding the depression level, most were classified within the low range (57.1%), while two individuals (9.5%) showed scores for severe depression. After intervention, all these profiles improved; the risk of PTSD reduced to 28.6%, the majority presented low symptoms of depression (81%), while the moderate group showed reduction from 33.3% to 19%. In addition, substantial shifts from high to moderate anxiety and perceived stress can be observed.

From the bivariate analysis, the psychometric questionnaires showed a reduction in all scores after intervention, with exception of the STAI-T, which did reduce even though without statistical significance (*p* = 0.147). Comparisons between PRE- and POST-intervention are shown in Table 4.

**TABLE 4:** PRE–POST psychometric questionnaire scores and corresponding statistical analysis.

Psychometric questionnaire	PRE	POST	Mean difference	*p*-value
VASS	66.57 (15.5)	31.95 (15.8)	34.62	<0.001 (a)
PCL5	35.71 (16.9)	24.43 (16.4)	11.29	0.001 (b)
PSS	19.05 (6.33)	15.43 (6.00)	3.62	0.023 (a)
STAI-S	30.33 (9.48)	18.81 (7.43)	11.52	<0.001 (a)
STAI-T	23.1 (10.6)	19.81 (8.90)	3.29	0.147 (b)
PHQ9	9.85 (5.82)	6.47 (4.80)	3.38	0.003 (b)

Key: Values represented as mean (SD). (a) Paired *t*-test. (b) Wilcoxon signed-rank. *p*< 0.05.

As exploratory analyses, we examined whether PRE–POST changes are influenced by age, BMI, physical activity, and daily practice. There are no significant findings. However, there is an effect related to the initial condition, i.e., those with high initial scores are more likely to show improvement in self-reported questionnaires (VASS: *p* = 0.003; PSS: *p* = 0.016; PCL5: *p* = 0.036; PHQ9: *p* = 0.016; STAI-S: *p* < 0.001), apart from STAI-T (*p* = 0.081).

#### 3.2.2 Chronic stress measurement

**FIGURE 4:**
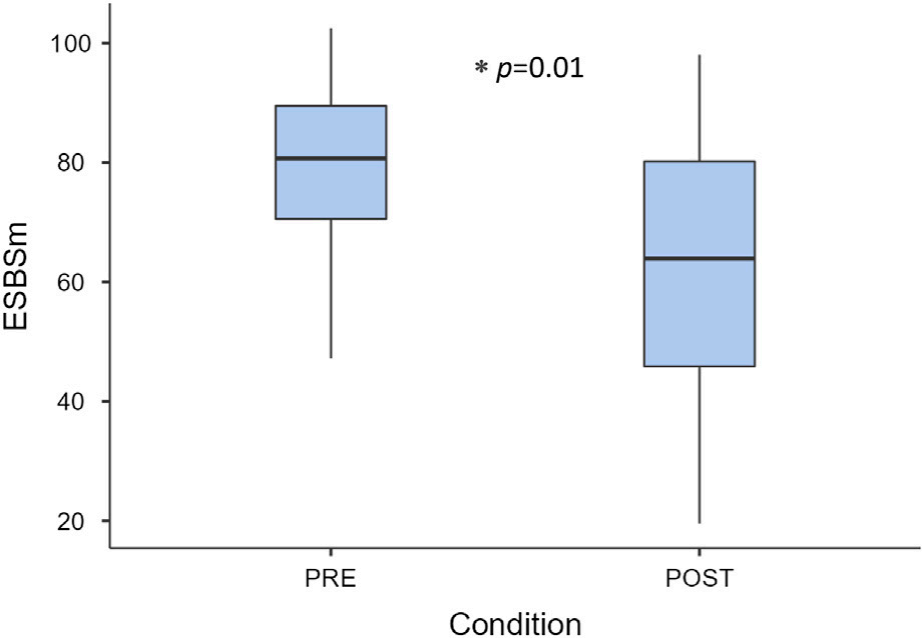
Chronic stress level at PRE- and POST-intervention measured by the multiparametric chronic stress model (ESBSm). Key: *p* < 0.05.

For PRE–POST comparisons, the baseline measurement (BM) was considered to mimic the conditions of the ESBSm chronic stress study. Figure 4 illustrates a reduction (77.6 vs. 62.8) found in the chronic stress level in POST-intervention when compared to PRE-intervention (paired *t*-test, *p* = 0.01), while the variance increased.

Similar to self-reported questionnaires, there is a significant effect related to the initial condition in the ESBSm results (*p* = 0.016), and no effect for age, BMI, or physical activity.

### 3.3 Secondary outcomes

#### 3.3.1 Physiological variables

The bivariate analysis revealed some significant differences (Table 5), such as a decrease in mean respiratory rate from 0.259 to 0.222 Hz (i.e., 15.5 to 13.3 breaths per minute) and an increase in HRV-SDNN, HRV-LF/HF, and HRV-LFn, with no differences for HRV-RMSSD.

**TABLE 5:** Electrophysiological measure comparisons and corresponding statistical analysis.

Parameters	PRE	POST	*p*-value
ECG			
HRV-HR, bpm	75.96 (9.48)	74.96 (10.01)	0.681 (a)
HRV-SDNN, s	0.044 (0.013)	0.051 (0.016)	0.027 (a)
HRV-RMSSD, s	0.012 (0.005)	0.012 (0.006)	0.801 (b)
HRV-sdsd, s	0.012 (0.005)	0.012 (0.006)	0.801 (b)
HRV-VLF, s^-2^	0.118 (0.087)	0.173 (0.123)	0.047 (b)
HRV-LF, s^-2^	0.226 (0.190)	0.288 (0.182)	0.078 (b)
HRV-HF, s^-2^	0.139 (0.120)	0.129 (0.120)	0.175 (b)
HRV-LF/HF ratio	204.77 (142.06)	413.15 (296.9)	0.003 (b)
HRV-LFn, nu	53.62 (13.86)	66.24 (12.65)	0.003 (a)
HRV-HFn, nu	46.38 (13.86)	33.76 (12.65)	0.003 (a)
PAT			
PAT, ms	240.96 (10.29)	242.57 (12.97)	0.657 (a)
stdPAT, ms	2.31 (1.52)	2.94 (3.29)	0.775 (b)
Resp			
RR, Hz	0.259 (0.07)	0.222 (0.09)	0.007 (a)
Pk, %	0.827 (0.04)	0.836 (0.06)	0.418 (a)
EDA			
Tonic, µS	−1.094 (0.285)	−0.948 (0.487)	0.199 (b)
Phasic, µS	0.038 (0.044)	0.099 (0.143)	0.012 (b)
aucPhasic, µS	2.259 (2.644)	5.940 (8.594)	0.012 (b)
EDASymp, µS	0.092 (0.170)	0.285 (0.627)	0.212 (b)
ST			
TFinger, °C	30.57 (4.06)	31.44 (2.49)	0.334 (b)
TGradient, °C	0.056 (0.058)	0.032 (0.073)	0.078 (b)
TPower, °C^2^	951.20 (220.41)	995.25 (151.05)	0.334 (b)

Key: Values represented as mean (SD). (a) Paired *t*-test. (b) Wilcoxon signed-rank. *p* < 0.05.

No significant changes in PAT and peripheral temperature parameters were observed. For EDA, the phasic component showed an increase (*p* = 0.012), whereas the average of the tonic component decreased POST intervention, although not significantly (*p* = 0.199).

#### 3.3.2 Acute stress measurement

Differences in physiological response measurement, i.e., between the baseline measurement (BM), stress exposure (SE), and recovery measurement (RM), were analyzed to visualize possible changes in the behavior of the signals during a stress-induced task. All mean values and respective p-values could be seen in Supplementary Table S1. The most relevant variables are illustrated on the following plots. Some of them were log-transformed for a better visual representation and were indicated with (log) in the axis title.

**FIGURE 5:**
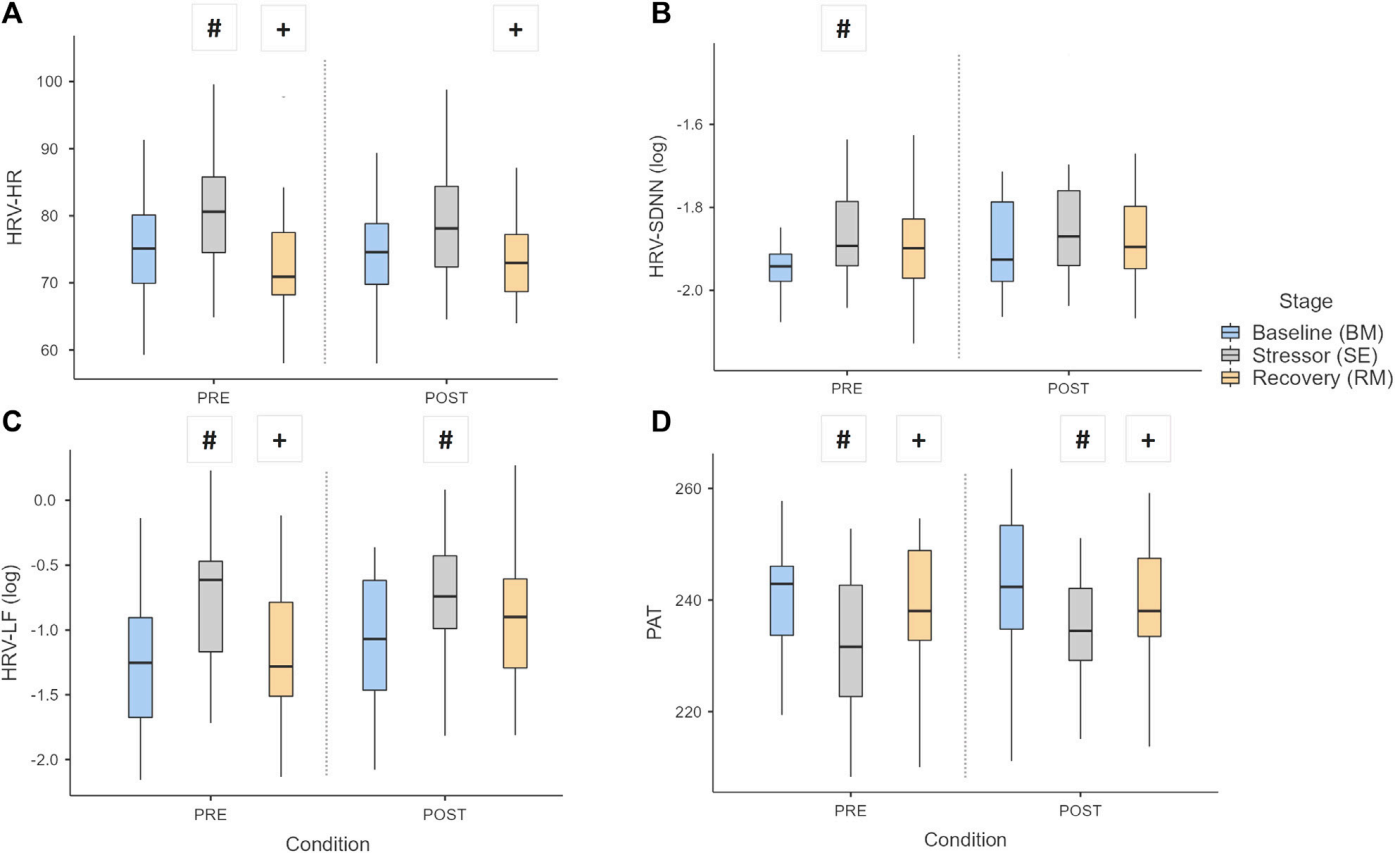
Boxplot of HRV parameters: **(A)** HR, **(B)** SDNN, **(C)** LF, and **(D)** PAT over the stages and conditions. Key: Light gray boxes show the significant differences between stages for each condition. (#) Differences between BM and the other stages (SE or RM). (+) Differences between RM and SE stages. *p* < 0.05.

The resting heart rate (HRV-HR) and pulse arrival time (PAT) showed significant differences in BM and RM stages when compared to SE (*p* < 0.001) under both conditions (Figures 5A, D, respectively). The HRV-SDNN changed its pattern, where BM differed from RM and SE stages in the PRE-intervention condition, which was not seen after intervention (Figure 5B). In the POST-intervention condition, SDNN values at baseline increased significantly (*p* = 0.027).

Figure 5C shows the behavior of the power in low-frequency band (HRV-LF), revealing differences between RM and SE at PRE-intervention, which did not occur after intervention.

**FIGURE 6:**
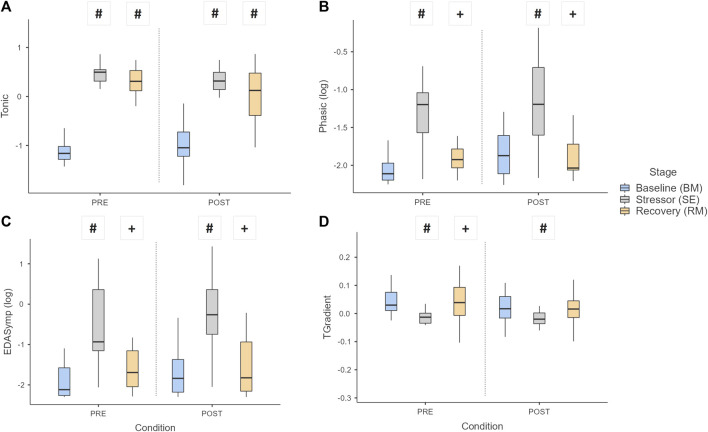
Boxplot of EDA and ST parameters: **(A)** Tonic, **(B)** Phasic, **(C)** EDASymp, and **(D)** TGradient, over the stages and conditions. Key: Light gray boxes show the significant differences between stages for each condition. (#) Differences between BM and the other stages (SE or RM). (+) Differences between RM and SE stages. *p* < 0.05.

Figure 6 shows the pattern of EDA and ST parameters. The tonic component (Tonic) of EDA showed differences between the BM and the other stages (*p* < 0.001) in both conditions (Figure 6A); furthermore, values at SE and RM reduced significantly POST intervention (*p* = 0.047 and *p* = 0.021, respectively). The phasic component (Phasic) and EDASymp differed between stages (*p* ≤ 0.001), but only Phasic showed difference in BM after intervention (*p* = 0.012). Regarding skin temperature, the gradient (TGradient) showed differences between the BM and RM stages in relation to the SE, in exception to RM vs*.* SE under POST condition (Figure 6D).

**FIGURE 7:**
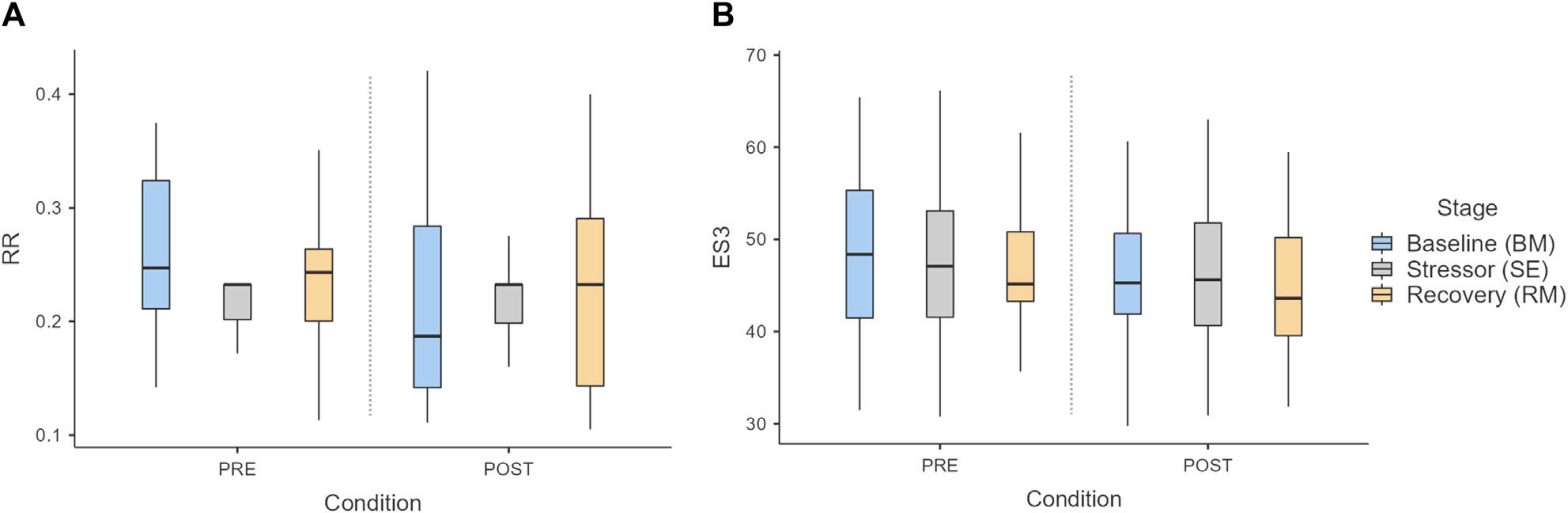
Boxplot of **(A)** respiratory rate (RR) and **(B)** ES3 acute stress model over the stages and conditions. Key: *p* < 0.05.

No differences related to respiration were observed between stages (Figure 7A), although the respiratory rate was overall reduced after the intervention, significantly only in BM (*p* = 0.007). The ES3, the model to assess acute stress, did not reveal differences between stages, although the scores were all lower after intervention (Figure 7B).

## 4 Discussion

Our study applied a new objective tool, the chronic stress multiparametric model (ESBSm), to assess the effectiveness of a 5-session HRV-BF protocol for HCWs presenting mental health symptoms due to their work in the frontlines of the COVID-19 pandemic. The improvement in mental health is corroborated by lower scores on self-reported psychometric questionnaires after intervention, reducing stress, risk of post-traumatic stress disorder, and symptoms of anxiety and depression. These results are consistent with those of previous studies that use similar instruments to assess the impact on mental health as published in this review (Di Nota et al., 2021), with some studies in conjunction with an enhancement of certain physiological biomarkers, such as heart rate variability. Thus, the results obtained in the procedure using the ESBSm support the hypothesis that a combined electrophysiological model provides useful and robust information about mental health state and could be considered to assess non-invasively changes over time.

Furthermore, the acute stress model (ES3), applied to compare physiological reactivity at baseline, stressor, and recovery measurements, showed an overall decrease after intervention, although nonsignificant. Unlike the chronic stress model, which aims to find differences in cumulative stress, this model investigates differences in acute physiological responses caused by stress-inducing tasks. As expected, there was an increase in the ES3 score when facing a stressor and a reduction in resting periods, in both conditions. This behavior indicates that the intervention did not affect the acute stress response in the studied sample.

Regarding the electrophysiological variables, a reduction in the basal respiratory rate is observed, which indicates that the participants were able to self-regulate their breathing, and this is helpful to stress management and to recover the autonomic balance. Additionally, this improvement in respiratory rate leads to better gas exchange and less chance of hyperventilation, which often occurs in disorders such as anxiety (Leyro et al., 2021). Our analysis related to respiration during the stress-inducing tasks were compromised once the tasks involved speech, so the data did not present the expected increase in respiratory rate which was observed in other types of stressors, like cold or heat exposure (Tipton et al., 2017).

An improvement in some HRV parameters, such as standard deviation of normal beats (SDNN) and power in low frequency (LFn), was also observed. These parameters are often used to assess the effectiveness of biofeedback intervention as they are considered ANS biomarkers. SDNN varies especially due to RSA, which is mediated by the parasympathetic system and precisely trained in HRV-BF, so an increase in this parameter indicates a successful intervention (Limmer et al., 2021). Furthermore, the LF power is influenced by both sympathetic and parasympathetic branches of the ANS, but reflects baroreflex activity in resting conditions (McCraty and Shaffer, 2015). Differences on the HRV-LFn may be related to the changes in respiratory rate since the low-frequency band (0.04–0.15 Hz) is influenced by breathing at 9 bpm or lower (Shaffer and Ginsberg, 2017). In this sense, the increase in LF power is expected after HRV-BF and corroborates the results shown in previous studies (Gross et al., 2016; Hasuo et al., 2020).

The pulse arrival time, which is an index that varies inversely with blood pressure, showed a decrease during the stress-inducing task when compared to baseline and recovery measurements. This pattern is in accordance with previous studies that demonstrated this reactivity in healthy students during a similar protocol for stress elicitation (Armañac et al., 2019; García Pagès et al., 2023). Concerning the skin temperature, no differences were observed after the intervention. All the parameters extracted from this signal showed the same expected pattern, reducing during the stress exposure and recovering afterward (Pisanski et al., 2018).

The variations of electrodermal activity (EDA) in our sample may correspond only to central nervous system modulation related to emotional and cognitive states of the individuals, once the environmental temperature interference can be discarded as the skin temperature did not vary over time (Sarchiapone et al., 2018). The phasic component of the EDA revealed differences after the intervention. This component, which corresponds to faster-changing activity, increased at baseline measurement, which indicates an augmented sympathetic arousal, on the contrary to what was expected after the intervention (Greco et al., 2021). We hypothesize that the participants anticipated their stress responses once they knew the elicitation tasks they were going to perform (i.e., Stroop test, arithmetic task, and description of feelings). Even so, it is important to highlight that both parameters extracted from the phasic component and the EDASymp were capable of distinguishing between the stressor of basal and recovery measurements. On the other hand, the tonic component of EDA showed an awaited pattern, increasing during the stress task, and a slower recovery time, seeming to accelerate slightly after the intervention, but still differing significantly from the baseline stage. During the stress exposure and recovery stages, the tonic level reduced significantly after intervention, which suggests an improvement in psychophysiological state and autonomic regulation (Marzi et al., 2021).

To summarize, the multiparametric chronic stress model comprises information of a combination of the most relevant physiological variables altogether, and their scores were in accordance with psychometric gold standards, therefore demonstrating to be a simple and objective tool to assess the effectiveness of a specific therapy. This procedure can be especially useful and easily applied in clinical practices, thus allowing to plan and properly combine different types of interventions for a more assertive treatment.

In the current study, the HCWs’ adherence was satisfactory, and they reported feeling motivated throughout the biofeedback protocol, which reinforces the feasibility of the intervention in this context and the importance of implementation by the health facilities as a preventive program. Moreover, HRV-BF is a proactive intervention that easily allows a multimodal measurement of electrophysiological parameters, ensuring the feasibility of the chronic stress model to assess its effectiveness and, more extensively, of other stress-reducing interventions.

Further research could replicate the proposed method in case-control studies and other interventions to confirm its efficacy for particular applications. To make the procedure even easier and unobtrusive, a remote wearable device may be used to monitor the mental status in real-time and to assess the effectiveness of interventions more extensively. A remote monitoring device would allow a longer follow-up and could be helpful in investigating other sources of stress and identifying protective and risk factors.

### 4.1 Limitations

One limitation of this study is that the recruited sample comprises only women; therefore, it is not possible to extrapolate our findings related to the effectiveness of HRV-BF for men. Second, the relatively small sample size (*n* = 21) is analyzed, which could be reflected in statistically nonsignificant results and thus increasing the risk of type II error (i.e., false negatives).

The PRE–POST experimental design does not prevent us from bias and limit the interpretation of the results. For this reason, we could not generalize the effectiveness of the applied intervention, which would be feasible in a case-control study, although, as an advantage, the present study design with paired comparisons avoids the influence of interpersonal variations of the electrophysiological variables. In order to use the multiparametric chronic stress model (ESBSm) proposed by Aguiló Mir et al. (2021), we assumed that the variability and severity of samples in both studies were similar and applied our standardized data to calculate the chronic stress level. The necessary corrections were also made in EDA parameters due to calibration problems related to the measuring device.

There was no follow-up to observe if the effects remained long-term after withdrawal of biofeedback. Further studies should be considered to assess the durability of the effects.

## 5 Conclusion

Our study demonstrates that a 5-session HRV-BF protocol is an effective intervention to reduce stress, the risk of post-traumatic stress disorder, and symptoms of anxiety and depression among our group of frontline HCWs who worked during the COVID-19 pandemic. Our findings support that the chronic multiparametric model based on electrophysiological variables provides relevant information about the current mental health status, being a reliable objective tool to assess the effectiveness of HRV-BF and may be useful for other stress-reduction interventions and contexts of burden in mental health. The variance increase in the model’s scores after the intervention indicates that the effectiveness of the intervention depends on each individual; therefore, a systematic measurement should be crucial for follow-up and treatment decisions. Further research should examine the robustness of these findings controlling possible confounders and, in addition, could investigate the reliability of wearables in providing feedback and monitoring physiological responses, which would constitute a more unobtrusive and easier-to-use instrument to reproduce the proposed procedure.

## Data Availability

The raw data supporting the conclusion of this article will be made available by the authors, without undue reservation.
