# Supplementary material for: Assessing effectiveness of heart rate variability biofeedback to mitigate mental health symptoms: a pilot study
Source: Front Physiol. 2023 May 10;14:1147260. doi: 10.3389/fphys.2023.1147260 (PMC10206049; doi:10.3389/fphys.2023.1147260)
Supplement: Supplementary file 1 [file Table1.DOCX]

Supplementary Material

**Supplementary Table 1.** Comparisons of electrophysiological variables and multiparametric acute stress model between stages (BM, SE and RM) by conditions (PRE and POST) and post-hoc with Tukey’s correction analysis.

| Parameters | Condition | Stages | | | Overall  p-value | Post-Hoc Tukey (p-value) | | |
| --- | --- | --- | --- | --- | --- | --- | --- | --- |
|  |  | BM | SE | RM |  | BM-SE | SE-RM | BM-RM |
| **Multiparametric acute stress model (ES3)** | | | | | | | | |
| ES3 | PRE  POST | 48.50 (8.83)  46.34 (8.43) | 47.06 (9.78)  45.82 (9.49) | 47.02 (8.03)  44.01 (7.04) | 0.618 (a)  0.397 (a) | 0.962  0.999 | 1.000  0.903 | 0.957  0.764 |
| **Electrocardiogram (ECG)** | | | | | | | | |
| HRV-HR, bpm | PRE  POST | 75.96 (9.48)  74.96 (10.01) | 80.55 (10.13)  78.22 (8.94) | 73.51 (8.80)  72.91 (6.08) | <0.001 (b)  <0.001 (a) | 0.002  0.056 | <0.001  <0.001 | 0.268  0.463 |
| HRV-SDNN, s | PRE  POST | 0.044 (0.013)  0.051 (0.016) | 0.057 (0.018)  0.059 (0.023) | 0.053 (0.021)  0.054 (0.017) | <0.001 (a)  0.228 (b) | 0.003  0.139 | 0.892  0.581 | 0.070  0.955 |
| HRV-RMSSD, s | PRE  POST | 0.012 (0.005)  0.012 (0.006) | 0.014 (0.006)  0.015 (0.009) | 0.017 (0.009)  0.014 (0.007) | 0.012 (b)  0.006 (b) | 0.544  0.175 | 0.244  0.955 | 0.003  0.650 |
| HRV-sdsd, s | PRE  POST | 0.012 (0.005)  0.012 (0.006) | 0.014 (0.006)  0.015 (0.009) | 0.017 (0.009)  0.014 (0.007) | 0.012 (b)  0.006 (b) | 0.545  0.176 | 0.243  0.955 | 0.003  0.651 |
| HRV-VLF, s^-2^ | PRE  POST | 0.118 (0.087)  0.173 (0.123) | 0.273 (0.203)  0.271 (0.224) | 0.110 (0.075)  0.110 (0.081) | <0.001 (b)  <0.001 (b) | <0.001  0.065 | <0.001  <0.001 | 0.999  0.458 |
| HRV-LF, s^-2^ | PRE  POST | 0.226 (0.190)  0.288 (0.182) | 0.439 (0.310)  0.437 (0.255) | 0.263 (0.221)  0.387 (0.313) | <0.001 (b)  0.180 (b) | 0.001  0.049 | 0.011  0.920 | 0.979  0.388 |
| HRV-HF, s^-2^ | PRE  POST | 0.139 (0.120)  0.129 (0.120) | 0.210 (0.166)  0.241 (0.278) | 0.213 (0.278)  0.146 (0.138) | 0.047 (b)  0.005 (b) | 0.372  0.035 | 1.000  0.110 | 0.326  0.997 |
| HRV-LF/HF ratio | PRE  POST | 204.78 (142.1)  413.15 (296.9) | 294.87 (175.7)  325.77 (189.8) | 197.44 (144.9)  475.08 (392.8) | 0.028 (b)  0.953 (b) | 0.731  0.756 | 0.661  0.203 | 1.000  0.930 |
| HRV-LFn, nu | PRE  POST | 53.62 (13.86)  66.24 (12.65) | 63.12 (11.01)  65.49 (10.22) | 54.87 (14.43)  69.47 (17.65) | 0.001 (a)  0.440 (a) | 0.021  0.999 | 0.067  0.755 | 0.998  0.881 |
| HRV-HFn, nu | PRE  POST | 46.38 (13.86)  33.76 (12.66) | 36.88 (11.01)  34.51 (10.22) | 45.13 (14.43)  30.53 (17.65) | 0.001 (a)  0.440 (a) | 0.021  0.999 | 0.067  0.755 | 0.998  0.881 |
| ***Pulse Arrival Time (PAT)** | | | | | | | | |
| PAT, ms | PRE  POST | 240.96 (10.29)  242.57 (12.97) | 231.16 (13.59)  233.85 (10.17) | 239.27 (10.96)  240.48 (11.29) | <0.001 (a)  <0.001 (a) | <0.001  <0.001 | <0.001  0.007 | 0.939  0.861 |
| stdPAT, ms | PRE  POST | 2.31 (1.53)  2.94 (3.29) | 4.30 (2.47)  4.12 (2.54) | 2.66 (1.36)  2.32 (1.46) | <0.001 (a)  <0.001 (b) | 0.017  0.127 | 0.079  0.010 | 0.992  0.561 |
| **Respiration** | | | | | | | | |
| RR, Hz | PRE  POST | 0.259 (0.07)  0.222 (0.09) | 0.233 (0.05)  0.212 (0.04) | 0.243 (0.07)  0.227 (0.09) | 0.118 (a)  0.563 (a) | 0.410  0.976 | 0.973  0.853 | 0863  0.998 |
| Pk, % | PRE  POST | 0.827 (0.04)  0.836 (0.06) | 0.770 (0.06)  0.768 (0.06) | 0.817 (0.06)  0.808 (0.06) | 0.001 (a)  <0.001 (a) | 0.008  <0.001 | 0.046  0.128 | 0.991  0.495 |
| **Electrodermal Activity (EDA)** | | | | | | | | |
| Tonic, µS | PRE  POST | -1.09 (0.28)  -0.95 (0.49) | 0.43 (0.27)  0.28 (0.32) | 0.33 (0.42)  0.05 (0.57) | <0.001 (b)  <0.001 (a) | <0.001  <0.001 | 0.955  0.447 | <0.001  <0.001 |
| Phasic, µS | PRE  POST | 0.04 (0.03)  0.10 (0.14) | 0.19 (0.11)  0.26 (0.19) | 0.05 (0.03)  0.06 (0.06) | <0.001 (b)  <0.001 (a) | <0.001  <0.001 | <0.001  <0.001 | 0.992  0.767 |
| aucPhasic, µS | PRE  POST | 2.26 (2.64)  5.94 (8.59) | 11.33 (6.45)  15.59 (11.37) | 3.19 (1.96)  3.80 (3.49) | <0.001 (b)  <0.001 (a) | <0.001  <0.001 | <.001  <.001 | 0.992  0.766 |
| EDASymp, µS | PRE  POST | 0.09 (0.17)  0.28 (0.63) | 0.74 (0.82)  0.91 (0.94) | 0.17 (0.25)  0.17 (0.21) | <0.001 (b)  <0.001 (b) | <0.001  <0.001 | 0.002  <0.001 | 0.996  0.961 |
| **Skin temperature (ST)** | | | | | | | | |
| TFinger, ºC | PRE  POST | 30.57 (4.06)  31.45 (2.49) | 30.41 (3.79)  30.80 (2.75) | 30.15 (3.63)  30.63 (2.91) | 0.552 (a)  0.016 (a) | 0.997  0.394 | 0.972  0.996 | 0.814  0.163 |
| TGradient, ºC | PRE  POST | 0.04 (0.06)  0.02 (0.05) | -0.03 (0.05)  -0.02 (0.05) | 0.04 (0.07)  0.02 (0.07) | <0.001 (a)  0.097 (b) | 0.003  0.035 | 0.023  0.128 | 0.985  0.995 |
| TPower, ºC^2^ | PRE  POST | 951.02 (220.41)  995.26 (151.06) | 938.93 (205.41)  956.37 (164.31) | 922.11 (195.96)  946.89 (170.47) | 0.470 (a)  0.014 (a) | 0.991  0.401 | 0.961  0.997 | 0.713  0.175 |

Key: Values represented as mean (SD). (a) ANOVA Repeated Measures. (b) Friedman. BM = Baseline measurement; SE = Stress exposure; RM = Recovery measurement; PAT is equivalent to PTT described in Aguiló and cols. (2020).
